# Supplementary material for: Characterizing the genetic diversity of the Andean blueberry (Vaccinium floribundum Kunth.) across the Ecuadorian Highlands
Source: PLoS One. 2020 Dec 7;15(12):e0243420. doi: 10.1371/journal.pone.0243420 (PMC7721170; doi:10.1371/journal.pone.0243420)
Supplement: S1 Table — (PDF) [file pone.0243420.s001.pdf]

S1 Table. Information for the 27 *V. floribundum* collection sites (CS) from 3 defined regions in the Ecuadorian Highlands.

Region	Collection site	Location	Province	Number of individuals	Elevation
Northern	CS1	La Cofradia	Carchi	3	3243-3260
	CS2	San Gabriel	Carchi	3	3405-3489
	CS3	El Angel	Carchi	4	3317-3402
	CS4	Cahuasquí	Imbabura	4	3588-3714
	CS5	Cuicocha	Imbabura	4	3080-3122
	CS6	Santa Lucía	Imbabura	3	2994-3062
	CS7	Mojanda	Pichincha	4	3596-3958
	CS8	Cayambe	Pichincha	3	3721-3885
	CS9	Lloa	Pichincha	4	3450-3546
	CS10	PNC	Cotopaxi	4	3550-3803
	CS11	Sigchos	Cotopaxi	3	3118-3147
Central	CS12	Quilotoa	Cotopaxi	4	4090-4131
	CS13	Tisandeo	Tungurahua	5	3542-3601
	CS14	Carihuairazo	Tungurahua	5	3627-3787
	CS15	Salinas Norte	Bolivar	3	4038-4062
	CS16	Salinas	Bolivar	3	3609-3670
	CS17	Cebapamba	Bolivar	4	3243-3285
	CS18	Quimiac	Chimborazo	7	3482-3758
	CS19	Cerro Abuga	Cañar	3	3029-3120
	CS20	Surimpalti	Cañar	4	3015-3066
Southern	CS21	San Miguel	Cañar	3	3044-3100
	CS22	Toreadora	Azuay	4	3929-3956
	CS23	Cajas	Azuay	3	4057-4098
	CS24	Cruces	Azuay	3	4154-4160
	CS25	Saraguro	Loja	3	2881-3008
	CS26	Santiago	Loja	3	2868-2911
	CS27	Podocarpus	Loja	4	3039-3067
