# Supplementary material for: Characterizing the genetic diversity of the Andean blueberry (Vaccinium floribundum Kunth.) across the Ecuadorian Highlands
Source: PLoS One. 2020 Dec 7;15(12):e0243420. doi: 10.1371/journal.pone.0243420 (PMC7721170; doi:10.1371/journal.pone.0243420)
Supplement: S2 Table — (PDF) [file pone.0243420.s002.pdf]

S2 Table. List of the 30 species-specific SSR markers designed for *V. floribundum*

Locus	Primer sequences (5'-3')	Motif	T °C
**Mo001***	**F:AACCTGTACAAGTCTACCCCTACCG**<br>**R: TAATAACAGAACATCAGTGCAAGGC**	**(TTCCTG)48**	**58**
**Mo002**	**F: CAAAATAACCCTCAAACACACACC**<br>**R: TTTATCATTATCCTACAGCGTCACC**	**(TTTGG)50**	**58**
Mo003	F: CTACCCATCCAACCACCTAACC<br>R: TAATAACAGAACATCAGTGCAAGGC	(ATGG)36	58
**Mo004**	**F: GAGGTATTGGAATCCTTGGATGG**<br>**R: CTCTTCCCCTCAACTCTTCCC**	**(TCC)33**	**58**
**Mo005**	**F: TAGAGATTCATCTCCATCCTTTTGC**<br>**R: GTCCATTAGGGTTCCAAAAGTGC**	**(ACC)33**	**58**
Mo006	F: GTTTGGAAGATCTCGCTGAGTAGG<br>R: CAGTAGAACCACTTACCCCTGTAGC	(ACC)27	63
**Mo007**	**F: GAAGCCTGGTCAGTCCTTTCC**<br>**R: CACTAGGAGTCTGACTTTCCTCTGC**	**(TGC)24**	**63**
**Mo008**	**F: ACTACCCTGCCACTCTCACTACC**<br>**R: CGGACCCAGAGTTAGGATAATACC**	**(ACC)27**	**63**
**Mo009**	**F: TATTCTTATGTTCGTCCTCGTAGGC**<br>**R: TTTCCTGCTAGCTGTTGTTGTAACG**	**(AGT)24**	**63**
**Mo010**	**F: TAGACAACCACTTTCTTTGGTTTCC**<br>**R: AATAAGTCTTGCTTTGTACCTTGCC**	**(TTC)30**	**63**
**Mo011**	**F: GCGAGAGTATTGGTGTTCATGC**<br>**R: CAGGTATAGATATACTGGGTTTTGAGG**	**(TTC)24**	**58**
Mo012	F: TGTTACGCTTATTACGTTGTGTTGG<br>R: TTCACAGTTGACTTGTTCTTATGCC	(ACC)24	60
Mo013	F: AGAGTACCATTTGGGTTAGTTTTGC<br>R: GACCAAAACAGTAGAAAACGACAGC	(TTC)33	60
Mo014	F: CTTTAAATGGAACCCCTCTTGTAGG<br>R: AATACATACAATCTCAGGCAAAGGG	(ATT)30	60
**Mo015**	**F: TAAATCCAAAAGGACAACTCCATCC**<br>**R: AACATGGGTTTAGCGTAGGAGACG**	**(TTC)27**	**60**
**Mo016**	**F: GAAGAAGAAATGGTGAGACAACTGC**<br>**R: AAGAAGATTGACTAGGGAGACATCG**	**(TTC)33**	**60**
Mo017	F: AAAACGTAGTTGGACAAACGATACG<br>R: GGTGGTTGTGGCCAAAATAGG	(ATT)42	60
**Mo018**	**F:ATTCGGGTATGGAGAGAGAAAGAGG**<br>**R: ACACCAACAAACCCGAAAATAACC**	**(TCC)27**	**60**
Mo019	F: AACCTGTGTAATCTCACCCACTACC<br>R: ATAGATGAGGTGCAACAAGAGTTGG	(AAC)33	58
**Mo020**	**F: CTACATTTTACCCGGTCACTTTTGC**<br>**R: CACACTAGTTACAAGAGCATTTTCCC**	**(ATT)33**	**60**
**Mo021**	**F: CATGGTTTGGTCTAGTTGATAACCC**<br>**R: GATGCTTCCTAGAGCCTTTATTGC**	**(TTC)33**	**60**
Mo022	F: CTTTAGAAACACGAAGTGACAGACC<br>R: TAGCTAATAGGTCAAGGGTTCAAGG	(TTC)30	60
Mo023	F: GACGACCAGAATAAAGAAGAGAAAGG<br>R: CTGAAGCCGACATATAGAAACTTGG	(ATT)24	60
**Mo024**	**F: TGTTGCTTCTTTTGTTTCCTACCC**<br>**R: TTAGAGTTCTAAGCCAACAAACTCG**	**(ATC)24**	**60**
**Mo025**	**F: GGTCAAAAGGAGGAGAATAATAGCC**<br>**R: TGTCTCTGCCCATTTTAATGTTACC**	**(TTC)54**	**60**
Mo026	F: AAGGGGCATCACTGTAATAGTATCG<br>R: GAACGAAATCATCGTCTTCACG	(TC)30	60
Mo027	F: CTTACCATGGTTGTAGCATTTTCCC<br>R: GTCCGTCAATTGTTTATGGATAAGG	(AT)20	60
Mo028	F: TGCTTTCATCTATTGACCTTATCGG<br>R: GGGTGTCGTATCAGTAATCTCTCG	(AC)18	60
Mo029	F: ACACCCTCAACTCAATACAGTGTGC<br>R: TGATGAGAATCCACCATTTTAGTGC	(TC)30	60
Mo030	F: TAGACAACCAAGCTCATTTTGATCG<br>R: CACTTAGAACAGGAACTCACCTTGC	(TC)18	60

* The 16 SSR markers used to genotype the Ecuadorian *V. floribundum* individuals are in bold type.
