# Supplementary material for: Characterizing the genetic diversity of the Andean blueberry (Vaccinium floribundum Kunth.) across the Ecuadorian Highlands
Source: PLoS One. 2020 Dec 7;15(12):e0243420. doi: 10.1371/journal.pone.0243420 (PMC7721170; doi:10.1371/journal.pone.0243420)
Supplement: S3 Table — Mean: the mean frequencies over the four genetic clusters. (PDF) [file pone.0243420.s003.pdf]

S3 Table. Estimate of null allele frequencies for each analyzed *V. floribundum* genetic cluster and SSR locus.

Locus	Cluster 1	Cluster 2	Cluster 3	Cluster 4	Mean
**Mo001**	0.089	0.131	0.088	0.091	0.100
**Mo002**	0.000	0.039	0.016	0.081	0.034
**Mo004**	0.275	0.215	0.017	0.117	0.156
**Mo005**	0.320	0.194	0.086	0.068	0.167
**Mo007**	0.224	0.142	0.052	0.048	0.117
**Mo008**	0.001	0.065	0.113	0.129	0.077
**Mo009**	0.352	0.174	0.215	0.106	0.211
**Mo010**	0.236	0.139	0.129	0.121	0.156
**Mo011**	0.001	0.126	0.048	0.000	0.044
**Mo015**	0.001	0.216	0.077	0.297	0.148
**Mo016**	0.342	0.160	0.171	0.000	0.168
**Mo018**	0.261	0.145	0.198	0.174	0.195
**Mo020**	0.176	0.150	0.104	0.224	0.163
**Mo021**	0.369	0.120	0.162	0.069	0.180
**Mo024**	0.330	0.089	0.091	0.082	0.148
**Mo025**	0.453	0.178	0.214	0.055	0.225

Mean: the mean frequencies over the four genetic clusters.
