# Supplementary material for: Characterizing the genetic diversity of the Andean blueberry (Vaccinium floribundum Kunth.) across the Ecuadorian Highlands
Source: PLoS One. 2020 Dec 7;15(12):e0243420. doi: 10.1371/journal.pone.0243420 (PMC7721170; doi:10.1371/journal.pone.0243420)
Supplement: S4 Table — NA (number of alleles); NPA (number of private alleles); AR (mean allelic richness calculated using rarefaction); HO (observed heterozygosity); HE (expected heterozygosity); and FIS (fixation index) are given for all 100 individuals genotyped at 16 SSR loci across 27 collection sites in three geographic regions. (PDF) [file pone.0243420.s004.pdf]

S4 Table. Summary of the diversity parameters for each collection site where *V. floribundum* individuals were collected.

Collection site	N_A	N_{PA}	A_R	H_O	H_E	F_{IS}
**CS1**	43	-	2.31	0.54	0.48	0.03
**CS2**	45	2	2.28	0.54	0.52	0.12
**CS3**	49	-	2.25	0.53	0.53	0.12
**CS4**	40	4	2.04	0.47	0.5	0.07
**CS5**	40	1	2.02	0.55	0.5	-0.05
**CS6**	36	1	1.83	0.46	0.42	0.11
**CS7**	42	3	1.97	0.34	0.46	0.33
**CS8**	28	2	1.31	0.14	0.21	0.4
**CS9**	29	1	1.37	0.23	0.27	0.19
**CS10**	42	2	2.05	0.42	0.46	0.15
**CS11**	37	-	1.78	0.35	0.38	0.14
**CS12**	26	1	1.23	0.07	0.21	0.78
**CS13**	53	5	2.15	0.46	0.5	0.2
**CS14**	60	6	2.54	0.58	0.6	0.17
**CS15**	42	1	2.16	0.46	0.42	0.08
**CS16**	42	1	2.23	0.58	0.49	-0.07
**CS17**	45	-	2.1	0.5	0.47	-0.01
**CS18**	65	5	2.49	0.55	0.6	0.1
**CS19**	39	1	1.99	0.52	0.46	-0.04
**CS20**	51	-	2.33	0.36	0.53	0.44
**CS21**	45	1	2.33	0.48	0.5	0.19
**CS22**	34	2	1.65	0.09	0.36	0.82
**CS23**	26	1	1.42	0.1	0.24	0.58
**CS24**	28	3	1.5	0.06	0.28	0.76
**CS25**	41	1	2.03	0.52	0.48	0.06
**CS26**	48	5	2.49	0.56	0.52	0.09
**CS27**	51	4	2.25	0.61	0.54	-0.02

N_A (number of alleles); N_{PA} (number of private alleles); A_R (mean allelic richness calculated using rarefaction); H_O (observed heterozygosity); H_E (expected heterozygosity); and F_{IS} (fixation index) are given for all 100 individuals genotyped at 16 SSR loci across 27 collection sites in three geographic regions.
