# Supplementary material for: Characterizing the genetic diversity of the Andean blueberry (Vaccinium floribundum Kunth.) across the Ecuadorian Highlands
Source: PLoS One. 2020 Dec 7;15(12):e0243420. doi: 10.1371/journal.pone.0243420 (PMC7721170; doi:10.1371/journal.pone.0243420)
Supplement: S5 Table — (PDF) [file pone.0243420.s005.pdf]

S5 Table. Values for estimating the optimum K from the analysis in STRUCTURE with an admixture model.

K	Reps	Mean LnP(K)	Stdev LnP(K)	Ln'(K)	|Ln''(K)|	Delta K
**3**	10	-4796.425	0.9500	283.750000	56.500000	59.473684
**4**	10	-4569.1750	1.0595	227.250000	117.475000	110.879760
**5**	10	-4459.4000	2.0510	109.775000	40.025000	19.514718
