# Supplementary material for: Characterizing the genetic diversity of the Andean blueberry (Vaccinium floribundum Kunth.) across the Ecuadorian Highlands
Source: PLoS One. 2020 Dec 7;15(12):e0243420. doi: 10.1371/journal.pone.0243420 (PMC7721170; doi:10.1371/journal.pone.0243420)
Supplement: S6 Table — (PDF) [file pone.0243420.s006.pdf]

S6 Table. Results of the analysis of molecular variance (AMOVA) performed for the *V. floribundum* genetic clusters that were identified in the population structure analysis (*P*=0.001).

**Source**	**Df**	**Sum Sq**	**Mean Sq**	**Est. Var.**	**%**
Between clusters	3	159.61	53.20	1.92	21.36%
Within clusters	96	677.47	7.06	7.06	78.64%
**Total**	99	837.08		8.98	100%
