# Supplementary material for: Characterizing the genetic diversity of the Andean blueberry (Vaccinium floribundum Kunth.) across the Ecuadorian Highlands
Source: PLoS One. 2020 Dec 7;15(12):e0243420. doi: 10.1371/journal.pone.0243420 (PMC7721170; doi:10.1371/journal.pone.0243420)
Supplement: S7 Table — (PDF) [file pone.0243420.s007.pdf]

S7 Table. Output for general linear model for the effect of the elevation over the heterozygosity.

Source	DF	Adj SS	Adj MS	F-Value	P-Value
Elevation	1	0.090684	0.090684	9.74	0.005
Error	25	0.232723	0.009309		
Lack-of-Fit	24	0.229523	0.009563	2.99	0.432
Pure Error	1	0.003200	0.003200		
Total	26	0.323407			
